# Radiographic views for hand fractures – call for three-view national UK guidelines – a quality improvement study

**DOI:** 10.1097/JS9.0000000000000381

**Published:** 2023-06-05

**Authors:** Clea Southall, Thessa R. Friebel, Sonya Gardiner, Dariush Nikkhah

**Affiliations:** Department of Plastic and Reconstructive Surgery, Royal Free Hospital, London, UK

**Keywords:** hand fractures, hand trauma, radiographs

## Abstract

A three-view radiographic examination (comprising of antero-posterior, oblique and lateral views) is crucial for the accurate assessment and subsequent decision-making in hand fracture management. The superiority of a three-view examination, compared to only two views, has been demonstrated by multiple studies, citing increased diagnostic accuracy and reduced rates of misdiagnosis. As such, the American College of Radiology (ACR) now recommends a standard three-view examination for finger and hand injuries; despite this, no formal guidance exists in the United Kingdom. Out of the 235 patients referred to our tertiary hand trauma unit with a confirmed hand fracture, less than half (45%) had three-view radiographic examination performed. Less than two-thirds (57%) of metacarpal fractures had three views available at assessment in our unit, with the lateral radiograph most commonly lacking (38%). Less than a third (30%) of phalangeal fractures had all three views, with the oblique view most commonly absent (64% of cases). Reviewed radiology protocols from six local hospitals were inconsistent; all recommended three views for suspected metacarpal fractures, but only two for suspected phalangeal injuries. Despite the superiority of a three-view examination and no additional cost of a third view, over half of the patients in this study lacked a three-view radiographic series. The authors would like to call for national published guidance advocating the use of three-view radiographic series in all patients with a high hand fracture suspicion (as defined by the presence of swelling, bruising and/or deformity) to reduce variability in local radiology hand fracture protocols and increase availability of three-view radiographs in the primary, secondary and tertiary settings.

## Introduction

Highlights

- Three-view radiographs increase diagnostic confidence and reduce misdiagnosis in hand trauma.
- Local protocols usually advise three views when a hand fracture is identified.
- But this is reliant on interpretation of radiographs in pressured emergency department.
- Additional radiation dose and cost for an additional hand radiograph are low.
- UK guidelines could standardise imaging protocols and aid hand fracture management.

Hand fractures are common, comprising up to 20% of all Emergency Department attendances. Early detection is crucial, relying on accurate clinical and high-quality radiographic examination^1^. Whilst many fractures may be managed non-operatively, up to 5% will require surgical intervention to restore range of motion and prevent complications^2,3^. Identifying complex fractures requiring surgical intervention is critical, as delays in diagnosis and missed fractures can be associated with long-term pain, severe disability and a prolonged time off work^1,4^.

Failure of fracture identification remains the most common diagnostic error in Emergency Departments^5^. Fractures of the extremities, in particular the phalanges, comprise a significant proportion of these missed fractures^6,7^. Whilst this is partially due to misreading of radiographs, literature suggests fractures are also missed when insufficient views in different dimensions are obtained^5,8^.

In established fractures, the radiographic examination plays a vital role in determining the appropriate management pathway. Three-view radiographs allow assessment of the fracture pattern, degree of angulation and displacement, which are all important factors guiding decision-making^9^.

The American College of Radiology (ACR) recommends a standard three-view examination for finger and hand injuries including antero-posterior (AP), oblique and lateral radiographs^9^. They conclude that a two-view radiographic approach is inadequate for accurately detecting fractures of the extremities including the hands and fingers. Unfortunately, at the time of writing, there is no formal guidance regarding the optimal imaging of hand fractures in the United Kingdom.

At our tertiary referral centre for Plastic and Hand surgery, we frequently encountered patients with hand fractures who had insufficient radiographic views performed at the referring unit. In conducting this study, we aimed to assess the magnitude of this issue and quantify which proportion of patients referred with a confirmed hand fracture had complete three-view radiographs available. Additionally, we reviewed the hand trauma radiology protocols from six referring hospitals, to check for variabilities and possible explanations for the incomplete series.

The Standards for Quality Improvement Reporting Excellent (SQUIRE) criteria were used to write this article^10^.

## Methods

The radiographic series of all phalangeal and metacarpal hand fracture patients presenting to the hand trauma clinic at the Royal Free Hospital between August 2020 and February 2021 were reviewed retrospectively. Patient demographics, referring hospital, type of fracture and radiographic views available were recorded for each case. Additional radiographs requested by our team in order to complete the radiographic series were also registered.

Across the 6 months period, 235 phalangeal and metacarpal fractures were included in this study. Volar plate avulsion fractures (*n*=12), bony mallets (*n*=12), tuft fractures (*n*=5), ulnar collateral ligament fractures (*n*=2) and central slip fractures (*n*=1) were excluded. Those excluded comprised a total of 12% (*n*=32) of the initial cohort.

All fracture and radiographic data were collected from internal hospital records and recorded into a predefined table in Microsoft Excel. Hand trauma radiology protocols from six local referring hospitals were requested and reviewed.

At the mid-point of the study, findings were communicated with the six radiology departments, requesting to improve compliance with local hand fracture protocols and consider changing their protocols to ensure an increased performance of all three views in hand fracture patients.

As this study was a quality improvement project, ethical approval was not required.

## Results

The 235 patients included in the study comprised 107 (46%) phalangeal and 128 (54%) metacarpal fractures. Only 45% of all patients had a complete three-view radiographic series available on arrival. All but one patient had an AP view; however, lateral radiographs were only available in 76% and oblique views in 69% (Table 1).

**Table 1:** Radiographic views available for all patients and after separation into pre-intervention and post-intervention (calling for radiology departments to adhere to and amend protocols).

	All patients, *n* (%)	Pre-intervention, *n* (%)	Post-intervention, *n* (%)
Number of patients	235 (100)	129 (54)	106 (45)
Three complete views	105 (45)	51 (40)	54 (51)
AP radiograph	234 (100)	128 (99)	106 (100)
Lateral radiograph	179 (76)	90 (70)	89 (84)
Oblique radiograph	161 (69)	90 (70)	71 (67)

AP, antero-posterior.

When stratified based on the anatomical bone fractured, only 30% of phalangeal fractures had all three views – AP views in all cases, a lateral view in 94% and an oblique view in 36% of cases. For metacarpal fractures, 57% had three views, 99% had an AP view, 62% a lateral view and 95% an oblique view (Table 2). Twenty-five patients with metacarpal fractures were sent for additional views by our team, compared to zero of the phalangeal fracture patients.

**Table 2:** Comparison of radiographs available for metacarpal and phalangeal fractures.

	Metacarpal, *n* (%)	Phalangeal, *n* (%)
Number of patients	128 (54)	107 (45)
Three complete views	73 (57)	32 (30)
AP radiograph	127 (99)	107 (100)
Lateral radiograph	79 (62)	100 (93)
Oblique radiograph	122 (95)	39 (36)

AP, antero-posterior.

Amongst the six local referring hospitals, there were variations in the views recommended by their radiology departments and low adherence to these protocols. For metacarpal injuries: all protocols advised AP and oblique views as standard, with a third view recommended only if there was a high index of suspicion or confirmed fracture. For phalangeal injuries: two protocols recommended AP and lateral views, two protocols all three views and two advised AP and oblique views with an additional lateral if specifically requested.

Communication with radiology departments at the mid-point of this study did not result in a significant improvement in the availability of three-view radiographs (Table 1).

## Discussion

On review of the current literature, three-view radiographs are used to increase diagnostic confidence and reduce misdiagnosis for both metacarpal and phalangeal fractures^4,11,12^. Multiple studies have demonstrated the superiority of a three-view radiographic examination compared to two views in assessment of traumatic fractures. De Smet *et al*.^11^ demonstrated that a three-view examination increased diagnostic accuracy and confidence in radiographic interpretation for traumatic hand, wrist and finger fractures compared to a two-view approach. They also demonstrated that an additional oblique view identified fractures that had otherwise been missed on the two views alone. Similarly, Jackson *et al*.^13^ demonstrated the addition of a third, oblique projection identified 4.4% more wrist fractures, when compared to two views, demonstrating the superiority of three-view radiographic examination in accurate assessment of fractures.

In this study, all referring unit protocols recommended a lateral view should be obtained if there was a high clinical suspicion or a confirmed metacarpal fracture. These are comparable with the hand fracture protocol of another major trauma centre in the UK^14^. However, only 57% of the metacarpal fractures in this study had all three views. For phalangeal fractures, the protocols were more diverse resulting in only 30% of these cases having three views available. The majority of phalangeal fractures had a lateral radiograph (94%), whilst only 62% of the metacarpal fractures had an available lateral view. Lateral radiographs play an important role in both the initial identification of metacarpal fractures (especially base of metacarpal fractures) and in determining their management^3^. Lamraski *et al*.^15^ demonstrated that the degree of metacarpal fracture angulation could only be accurately assessed on the lateral view and, conversely, was overestimated when using the oblique view alone. Consequently, it has been argued that the degree of angulation can only be reliably assessed by drawing lines on the lateral radiograph specifically^16^. The 25 patients re-imaged at our centre were all metacarpal fractures, sent to obtain the missing lateral radiograph. Three views, in particular a true lateral view, enable surgeons to fully assess fracture patterns, degrees of angulation and the presence of subluxation. This information guides decision-making as to whether operative intervention is required and, if so, what fixation technique should be employed.

Whilst three views may not be essential for all hand fractures cases, we believe based on the published literature, having three views as standard is the safest approach. National UK three-view guidelines would also remove the reliance on interpretation of radiographs in pressured emergency department, to determine which views are ought to be taken. A recent study performed in a major trauma centre in the UK has also called for a standardised, three-view radiographic examination of hand trauma^14^. Hassan *et al.* compared the sensitivity and specificity of different radiographic views, demonstrating that the oblique and lateral views both have good negative predictive values, increasing confidence in excluding a fracture. Importantly, they also looked at the accuracy of diagnoses between Orthopaedic trainees and Emergency Nurse Practioners (ENPs). They found that the lowest accuracy was observed in the ENP group, where 22% of injuries were missed. Increased pressure on Emergency Departments has led to a more prominent role of the ENPs in assessing minor hand injuries and trauma. Confidence in assessment and diagnosis of hand fractures will likely vary between individuals, based on expertise and education. Standardisation of radiographic examination should therefore be complimented by increased education on reading radiographs, to improve diagnostic accuracy and reduce cases of misdiagnosis^14^.

At our tertiary centre, there is no additional cost for a third radiographic view when performed with the other two views. However, even where there is an additional cost per radiograph, this should be offset by the cost of incorrectly managed patients especially with increased litigation after hand and wrist trauma^17,18^. For example, a missed Carpometacarpal fracture–dislocation received £177 814 in compensation payout^14^. Furthermore, the radiation dose for an extra radiographic view in the hand is only less than 0.001 mSV (equivalent to less than 1 day of background radiation exposure), so we believe the benefits of conducting three-view radiographs in hand injuries with a high suspicion of fractures far outweigh the risks^19^.

With multiple studies demonstrating the diagnostic superiority of three-view over two-view radiographs in hand fracture identification^4,5,8,9,11–16^, we were surprised by the differing radiology protocols for hand trauma series in the six different radiology units reviewed. Failure to improve availability of three-view series after communication with radiology departments clinical leads was not unexpected. We believe national guidelines would have a much greater impact and could standardise imaging of hand fractures across the UK.

The main limitation of our study is the lack of information on whether three-view radiographs were indeed superior to two views for fracture identification at initial presentation and management in our unit. As only the final diagnosis and management plan were documented by our clinical team, we do not know whether this was altered after obtaining all three radiographic views. Although it was not within the remit of this study, we appreciate the correlation with patient-related outcomes such as delays in diagnosis, changes in management or the effect on functional outcomes or patient satisfaction would have been desirable. However, given the fact that multiple studies have already demonstrated the superiority of three views, the aim of our study was primarily to assess the variability in protocols and evaluate the scale of missing radiographs in fracture patients referred into tertiary care.

Moving forward, a prospective cohort study including all hand trauma patients presenting to the emergency department could provide us with valuable information with regards to diagnostic accuracy when comparing fracture identification on two or three radiographic views. Each hand trauma patient with a high fracture suspicion would have an AP, lateral and oblique view performed. On initial assessment, only two radiographic views would be available, and reviewers would be asked to identify any possible fractures and draw up a management plan. This would be followed by a second assessment with all three radiographic views provided. Data generated by this follow-up study could provide some evidence to address the limitations of the currently presented study.

## Conclusion

This study has demonstrated that a significant proportion of referred hand fracture patients received an incomplete radiographic examination prior to assessment in our clinic. We find this concerning as literature has shown a higher incidence of false-negative diagnoses in emergency department if only two-view radiographs are available. Additionally, this study highlights that whilst referring hospitals recommend three-view examination for suspected metacarpal fractures, only two of the six protocols recommend three views for suspected phalangeal injuries.

We would like to call for national published guidelines advocating the use of three-view radiographic series in all hand trauma patients with a high suspicion of fractures, to reduce variability and improve availability. This should be complimented by increased training of all healthcare professionals assessing hand trauma, ensuring adequate understanding of the importance of a three-view radiographic examination. Increased education and standardised protocols should reduce the incidence of misdiagnosis, avoid extra time, costs and workload for additional views requested at a later time, and aid vital decision-making with regards to hand fracture management.

## Ethical approval

No ethical approval was required as this was a quality improvement study.

## Sources of funding

This research did not receive any specific grant from funding agencies in the public, commercial or not-for-profit sectors.

## Author contribution

All authors (C.S., T.R.F., S.G. and D.N.) have contributed to all aspects of the study – design, data collections, data analysis and writing.

## Conflicts of interest disclosure

There are no conflicts of interest.

## Guarantor

Clea Southall is the guarantor for this study.

## Data availability statement

Data available on request – raw data available in Microsoft Excel datasheet.

## Provenance and peer review

Not commissioned, externally peer-reviewed.
